# Energy Harvesting from Upper-Limb Pulling Motions for Miniaturized Human-Powered Generators

**DOI:** 10.3390/s150715853

**Published:** 2015-07-03

**Authors:** Jeongjin Yeo, Mun-ho Ryu, Yoonseok Yang

**Affiliations:** 1 Healthcare Engineering, Chonbuk National University, Deokjin-dong Jeonju 664-14, Korea; E-Mail: yeojjin85@jbnu.ac.kr; 2 Biomedical Engineering, Chonbuk National University, Deokjin-dong Jeonju 664-14, Korea; E-Mails: mhryu@jbnu.ac.kr

**Keywords:** human-powered generator, pulling energy harvester, human kinetics, flywheel magnet rotor, coreless coil

## Abstract

The human-powered self-generator provides the best solution for individuals who need an instantaneous power supply for travel, outdoor, and emergency use, since it is less dependent on weather conditions and occupies less space than other renewable power supplies. However, many commercial portable self-generators that employ hand-cranking are not used as much as expected in daily lives although they have enough output capacity due to their intensive workload. This study proposes a portable human-powered generator which is designed to obtain mechanical energy from an upper limb pulling motion for improved human motion economy as well as efficient human-mechanical power transfer. A coreless axial-flux permanent magnet machine (APMM) and a flywheel magnet rotor were used in conjunction with a one-way clutched power transmission system in order to obtain effective power from the pulling motion. The developed prototype showed an average energy conversion efficiency of 30.98% and an average output power of 0.32 W with a maximum of 1.89 W. Its small form factor (50 mm × 32 mm × 43.5 mm, 0.05 kg) and the substantial electricity produced verify the effectiveness of the proposed method in the utilization of human power. It is expected that the developed generator could provide a mobile power supply.

## 1. Introduction

Countless mobile devices, such as MP3 players, digital cameras, smart phones, tablet PCs, and smart watches have been developed during the last several decades. More and more people are spending time sharing their lives and concerns through the seamless mobile network using those devices. The substantial decrease in power consumption of electronic components and huge improvements in battery technology were expected to help people use these devices for an extended period of time. However, increased battery capacity was counteracted by new functionalities, and the devices still suffer from limited usage time due to the additional power consumption from added components and operations. Consequently, there is no other way for people to ensure incessancy of those anytime-anywhere necessities but to carry extra batteries for travel, outdoor use, and emergency conditions [1]. Mobile users are often observed wandering around hunting for any available power grid connections such as wall sockets. The continuity of power supply is the most essential condition for a truly seamless mobile environment.

Many technologies have been proposed to convert the energy that can be obtained from surroundings into electricity. The human body has also been considered as an excellent platform for applying such technologies, since the body contains a lot of ambient energy [2]. There have been many attempts to supply power to mobile devices in real-time using the energy generated by the human body [3,4,5,6,7,8,9,10]. They include shoe-mounted generators [3,4,5], knee-mounted generators [6], energy harvesting on a backpack [7,8] and harvesting energy from breathing [9,10]. They accomplished huge improvement in economic and effective use of human motion by designing sophisticated structures mounted on unique locations on the human body or wearables. Some of them even suggested that waste energy in human motion can be utilized [6,7] to generate electricity. However, since their implementation require delicate manufacturing depending on the mounting configuration, there are still many problems to be tackled for practical development and further commercialization. Also, the level of output power and operating schemes are not yet suitable for an instantaneous self-powering generator. On the contrary, there are many commercial portable human-powered generator products on the market [11,12]. They are often equipped with internal batteries and additional functionalities such as flashlights, of AM/FM radios in order to extend their application area. Some of them have enough output capacity to supply mobile devices. The major concern of those conventional self-generators is to generate as much power as possible by intensifying the magnetic field density while enduring the large handling torque of the crank [13]. There has been very little consideration about the crank rotation kinetics in conjunction with human limb motion. As a consequence, the energetic cost of the human upper limb motion needed to power the existing self-generators through cranking is too high. These products are not used as much as expected in daily lives except for getting their internal battery charged from an external power grid as extra backup for other mobile devices. Therefore, they often unfavorably impress users with a feeling that generating electricity by human power is a difficult, inconvenient, and therefore obsolete idea.

Nevertheless, the human-powered self-generator still provides the best solution for individuals who are in need of an immediate and reliable power supply since it is less dependent on weather conditions and occupies smaller volumes than any other renewable energy source. With much improved designs of practical mechanical power transmission and electromagnetic configurations as well as simple operating schemes and structures, it should be possible to realize a practical portable human-powered generator.

This study proposes a viable human-powered generator designed to extract mechanical energy from an upper limb pulling motion, which is more adequate for consistent portable use than conventional hand-cranking in the aspects of human motion economy, the efficiency of human-mechanical power transfer, and electric energy conversion. Its prototype is implemented by a new combination of a coreless axial-flux permanent magnet machine (APMM) with a flywheel magnet rotor and a one-way clutched power transmission system to derive effective power from the human kinetic energy source. Experimental measurements using the developed prototype have verified that the proposed pulling energy harvester was successful in generating substantial amounts of electricity with higher energy conversion efficiency than conventional crank generators by effectively obtaining human power from a pulling motion. Some future possibilities are suggested based on the results of this study.

## 2. Materials and Methods

### 2.1. Human Motion for Power Production

**Figure 1:**
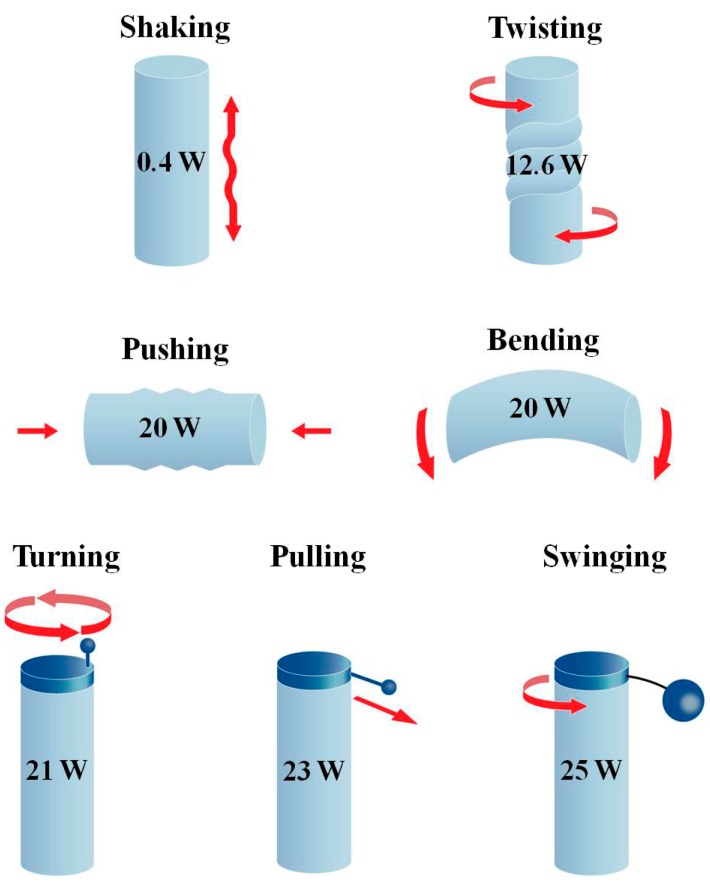
Power production from various upper limb motions.

Figure 1 shows that the upper limb can implement a wide range of dynamic motions with precise control [13].

This is one of the reasons that the majority of portable self-generators are designed to be operated by upper limb motion, not by motions of the lower limbs or any other parts of the human body. Theoretically, the energy produced by upper limb motion is enough to operate a small electronic device through a generator and power conditioning circuits for proper conversion and regulation [13]. From the previous studies on various upper limb motions, it has been learned that turning, pulling, and swinging can produce mechanical power greater than 20 W [13].

Though the swinging motion produces the largest amount of power, it is not considered suitable for a portable self-generator since it requires rather heavy orbiting component and extra space for safe and stable swinging. The pulling and turning motions do not require large space or heavy weights; they also produce a significant amount of mechanical power. However, the crank should be long enough for easy turning, which determines the volume of the device when miniaturized, while a pulling string can be concealed by being wound-up around a small bobbin. Therefore, pulling was considered the best candidate for a dynamic energy source of the portable self-generator. Moreover, it will be shown that pulling is better in overall efficiency of converting human power into electricity by reducing kinetic losses, not only in transferring the mechanical power of limb motion to the generator but also in producing the limb motion, as shown below.

#### 2.1.1. Mechanical Power Transfer into the Generator

**Figure 2:**
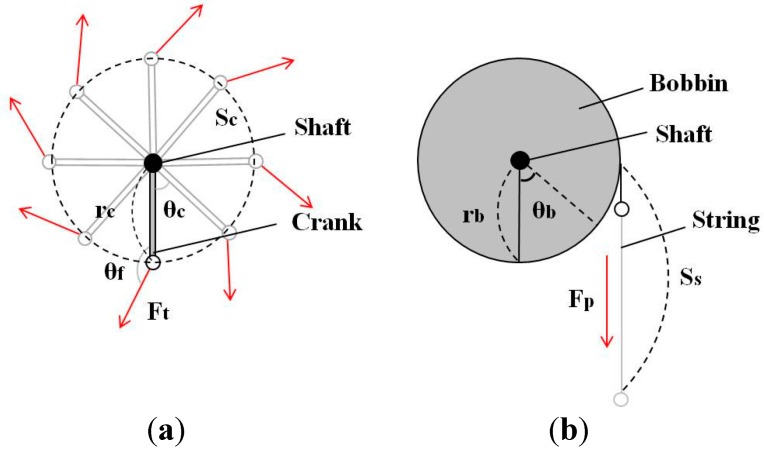
Analytic models of operation by human upper limb motion. (**a**) Turning; (**b**) Pulling.

Analytic models of turning and pulling operations are illustrated in Figure 2 to compare their efficiency in transferring mechanical power from upper limb motion to the generator shaft. Detailed parameters of each model are listed in Table 1. The turning torque *τ_c_* applied on the shaft in Figure 2a is represented by Equation (1):

(1) $$\tau_{c}=r_{c}\cdot F_{t}\cdot \sin\theta_{f}$$

The mechanical work transferred to the shaft for rotating the shaft by *θ* can be obtained by Equation (2):

(2) $$W_{t}=\int_{0}^{\theta }\tau_{c}\cdot d\theta_{c}=\int_{0}^{\theta }r_{c}\cdot F_{t}\cdot \sin\theta_{f}\cdot d\theta_{c}$$

Next, the kinematic work done by human limbs to move the distal end of the crank by *S* is calculated as shown in Equation (3) by using the relationship between the incremental displacement *dS_c_* and incremental angle *dθ_c_* represented by Equation (4):

(3) $$W_{t\_human}=\int_{0}^{S}F_{t}\cdot dS_{c}=\int_{0}^{\theta }F_{t}\cdot r_{c}\cdot d\theta_{c}$$

(4) $$dS_{c}=r_{c}\cdot d\theta_{c}$$

It is clear that the work transferred to the shaft rotation is always smaller than or equal to the work done by the human limb motion as shown in Equation (5):

(5) $$W_{t}\leq W_{t\_human}$$

Only precise control of both the limb motion and the direction of force acting at the distal end of the crank handle along the circular trajectory could fully transmit the motion power to the shaft. This implies that turning has an unavoidable energy loss in delivering human mechanical power to the self-generator.

On the contrary, for the pulling model in Figure 2b, the mechanical work that rotates the shaft by *θ* is obtained by Equation (6), where *τ_b_* is the torque on the shaft given by Equation (7):

(6) $$W_{p}=\int_{0}^{\theta }\tau_{b}\cdot d\theta_{b}=\int_{0}^{\theta }r_{b}\cdot F_{p}\cdot d\theta_{b}$$

(7) $$\tau_{b}=r_{b}\cdot F_{p}$$

Like Equation (3), the kinematic work done by the human limb to pull the string by *S* is represented by Equation (8) using the relationship between the length of the string drawn by pulling *dS_s_* and the radius of the bobbin *r_b_* shown in Equation (9):

(8) $$W_{p\_human}=\int_{0}^{S}F_{p}\cdot dS_{s}=\int_{0}^{\theta }F_{p}\cdot r_{b}\cdot d\theta_{b}$$

(9) $$dS_{s}=r_{b}\cdot d\theta_{b}$$

Therefore, in the case of pulling motions, the mechanical work delivered to the shaft rotation is equal to the mechanical work done by human limb motion as shown in Equation (10).

(10) $$W_{p}=W_{p\_human}$$

**Table 1:** Symbols in Figure 2.

	Symbols	Units	Variables
Turning	*r_c_*	m	Length of crank handle
	*S_c_*	m	Displacement of crank-end
	*θ_c_*	rad	Angular displacement of crank handle
	*F_t_*	N	Force applied by upper limb
	*θ_f_*	rad	Angle between the crank handle and the F_t_
Pulling	*r_b_*	m	Radius of bobbin
	*S_s_*	m	Displacement of string
	*θ_b_*	rad	Angular displacement of bobbin
	*F_p_*	N	Force applied by upper limb

#### 2.1.2. Economy of Human Limb Motion

As illustrated in Figure 2, the acting point of force in turning motions should follow a circular trajectory, while it follows a linear trajectory in pulling motions. In the kinetic view of human musculoskeletal motion, the circular motion of the upper limb is much more complicated than the linear one since it requires precise control of multi-joint arm movements. More importantly, to maintain the circular trajectory of multi-joint arm movements while managing the orientation of force at the end-point correctly, *i.e.*, perpendicular to the crank handle, agonistic and antagonistic muscles must be recruited concurrently, which yields tonic contraction of the muscles [14]. This greatly increases the energetic cost of human limb motion more than necessary for the shaft rotation, and consequently decreases the economy of human limb motion. Finally, it is more difficult to maintain a steady and stable circular motion if the turning motion is faster.

Hence, if the upper limb turning motion and the direction of force are not accurately controlled, the entirety of produced power will not be transferred to the generator as shown in Equation (5). Still, if they are to be exactly controlled, there will be unavoidable metabolic energy waste due to the tonic contraction of the agonistic and antagonistic muscles.

Additionally, it has been shown that the human central nervous system prefers a trajectory in which the joint-torque is minimally changed in planning and controlling the upper limb motion [14,15]. Pulling is more intuitive and efficient from a neurological point of view because it requires less change in joint-torque during limb movements than turning. Therefore, it is analytically verified that the pulling motion is more suitable for a miniaturized self-generator than turning in terms of the efficiency of mechanical energy transfer, human energy economy, and neurologic preference.

Nevertheless, the pulling motion has rarely been used in conventional self-generating products. The turning motion has largely been adopted in most portable self-generators. This can be attributed to the fact that the rotational motion of the crank fits well in accordance with the motion of the rotor-stator pair inside the generator. More importantly, the turning of a sufficient length of crank can apply enough torque on conventional electromagnetic generators to exceed the cogging force, which is an adverse effect caused by using metal or ferromagnetic cores for intensifying magnetic flux density.

If the radius of the bobbin is the same as the length of the crank handle in Figure 2, enough torque could be applied to the generator easily, but portability would be diminished. If a smaller bobbin must be used to miniaturize the generator, it is hard to apply a large enough torque to overcome the cogging and to obtain fast rotation of a magnet rotor. In this case, the power derived from the pulling motion and put into the generator is far less than its maximum possible value shown in Figure 1. As a consequence, the generated electric power is inevitably small. The pulling motion applied with a small bobbin needs a specially configured generator that enables the effective extraction of human power from the low torque operation to generate substantial electricity.

### 2.2. Design and Implementation of the Proposed Wearable Pulling Generator

**Figure 3:**
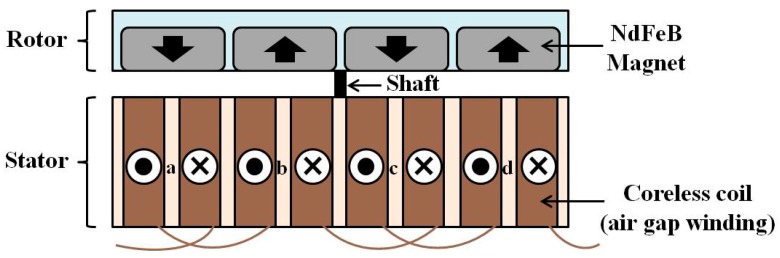
Electromagnetic coupling structure of the proposed APMM.

Figure 3 shows the APMM scheme proposed in this study in which a neodymium (NdFeB) magnet rotor and the coil stator are coupled in an axial direction for better miniaturization [16,17,18]. All 12 magnets were positioned in the disc rotor along its edge with alternating polarities. Every two coils were electrically paired to double the induced voltage when moving magnetic field changes its polarity in the midst of each paired coil.

**Figure 4:**
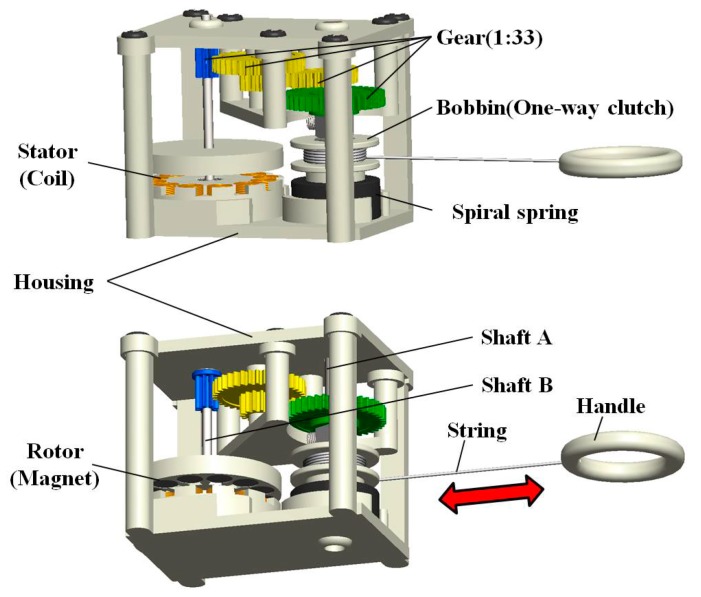
3-D designed structure of the pulling energy harvester.

In Figure 1, it is supposed that the high speed rotating body is a key to the large amount of power production from the pulling motion. Therefore, since it is essential to decrease the torque required for rotating the magnet rotor, air-cored coils replaced the usual metal-cored coils in the APMM to eliminate the cogging torque. The 3-D detail of a designed pulling energy harvester is provided in Figure 4, which shows the arrangement of its components. The small bobbin is mechanically linked to the magnet rotor via a one-way clutch and high-ratio gear. A metal string is wound on the bobbin and a spiral spring connects the bobbin to shaft A for rewinding the string. The one-way clutch enables the pulling force to produce a unidirectional rotation of the magnet rotor by disengaging the bobbin from the gear during the rewinding intervals, during which the bobbin rotates in the opposite direction to rewind the string. Additionally, it is expected that the removal of cogging will allow the inertial mass augmented by 12 magnets placed in the disc rotor to serve the purpose of preserving its rotation during the rewinding intervals between consecutive pulling cycles, similar to the flywheel employed in large wind turbine generators.

**Figure 5:**
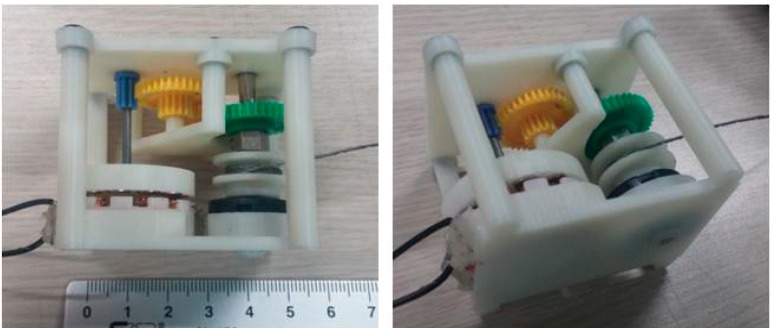
Prototype of the developed pulling energy harvester.

Figure 5 shows the developed prototype of the pulling energy harvester. Most of the components, including the rotor, frame, and housing were manufactured by 3-D printing with acrylonitrile butadiene styrene (ABS). However, the high-speed gear was constructed by assembling toothed wheels recycled from toy cars for enhanced durability. The spiral spring and metal string were also recycled from a commercial-grade retractable key chain.

The prototype has a relatively small form factor with a weight of 0.05 kg, even though it was constructed with many off-the-shelf components. It can be carried and gripped easily by one hand. More detailed parameters are listed in Table 2.

**Table 2:** Specific parameters of the developed prototype of pulling energy harvester.

	Variables (Units)	Value
Mechanical parameters	Volume (mm^3^)	50 × 32 × 43.5
	Weight (kg)	0.05
	Length of string (mm)	180
	Bobbin radius (mm)	5.65
	Gear ratio	1:33
Electromagnetic parameters	Rotor diameter (mm)	30
	Rotor thickness (mm)	5
	Magnet diameter (mm)	5
	Magnet thickness (mm)	3
	Magnet surface field (gauss)	3850
	Number of rotor magnets	12
	Stator diameter (mm)	30
	Stator thickness (mm)	8
	Outer diameter of solenoid coil in stator (mm)	1
	Inner diameter of solenoid coil in stator (mm)	8
	Wire diameter of solenoid coil in stator (mm)	0.15
	Number of turns in solenoid coil	380 (each coils)
	Number of solenoid coils in stator	12
	Total resistance of the solenoid coils (Ω)	36

### 2.3. Experiments

**Figure 6:**
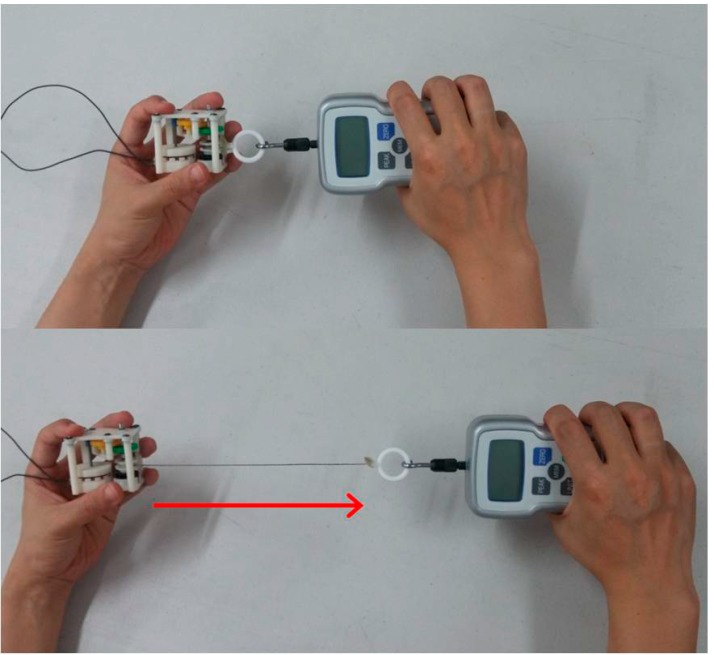
Pulling experiment using the developed prototype of pulling energy harvester and push-pull gauge.

**Figure 7:**
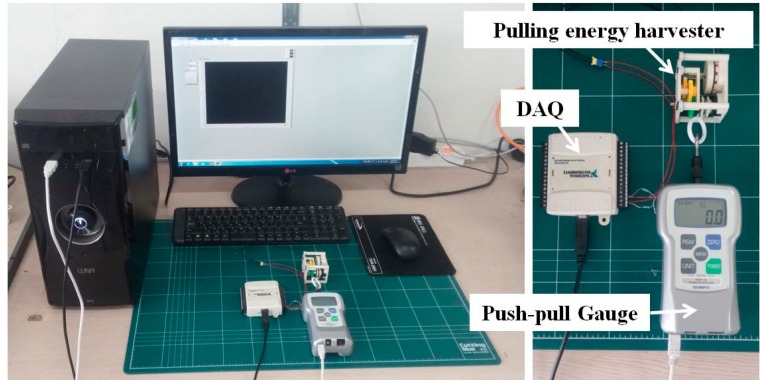
Experimental set-up for the simultaneous measurement of mechanical input and electric output power.

In order to verify the performance of the proposed pulling energy harvester, both the pulling force and the generated electric voltage were measured. As shown in Figure 6, the pulling force of the upper limb was measured at the end of the string by using a push-pull gauge with data logging capability. Concurrently, the generated electric voltage was measured with a resistive load connected to the output terminals of the generator by using a data acquisition device. The resistance was set to 36 Ω, which was the same value as the internal resistance of the generator determined by the coil wires. Figure 7 shows the experimental setup. The measuring experiment was performed for 10 consecutive pulling cycles. The mechanical input power and corresponding electric output power were calculated from the recorded data. The entire time-profiles of the force and the voltage were recorded not only to compute average power but also for an in-depth analysis of the dynamic characteristics of its operation.

## 3. Results and Discussions

**Figure 8:**
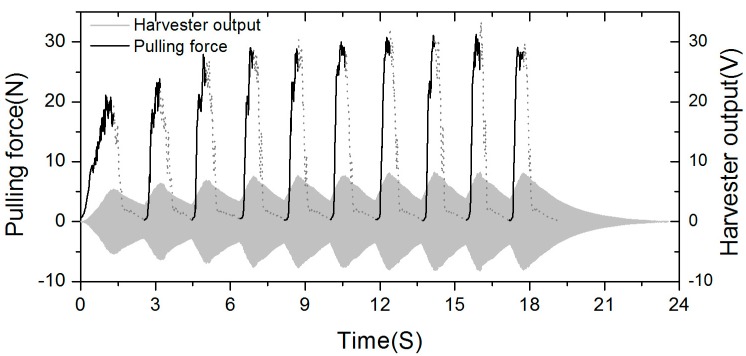
The recorded waveforms of pulling force and output voltage.

Figure 8 shows the recorded waveforms of input force and output voltage for 10 consecutive pulling cycles. A pulling force with a maximum of 30 N was applied to the string with a rate of 0.56 cycles/s. The generated voltage showed a maximum of 16 V_pp_ with the matching resistive load. The mechanical input work was calculated from the measured time profiles of the force and the displacement of string by using Equation (9). Since the repetitive voltage waveforms are in accordance with the revolution of the rotor, the displacement could be accurately obtained by examining the number of voltage peaks, gear ratio, and bobbin radius as follows. The 12 magnets positioned on the rotor with alternating polarities generate six positive voltage peaks when the rotor revolves one time. If the bobbin revolves one time, the rotor is turned 33 times by the gear ratio (1:33). Thus, 198 positive voltage peaks occur when the bobbin revolves one time. Since the circumference of the bobbin adopted in our prototype energy harvester is about 35.5 mm, a gap between two adjacent positive voltage peaks in electrical output waveform, which is defined as peak distance, *dD_p_,* corresponds to 0.18 mm displacement of pulled string, Then, the displacement *dS_s_,* within specific period of time can be obtained by measuring *dD_p_* as shown in Equation (11):

(11) $$dS_{s}=0.18\cdot dD_{p}$$

However, it is important to note that the mechanical input power is calculated by averaging the mechanical work only over the active pulling phase, *i.e.*, excluding rewinding phase, in order to make it possible to directly compare it with theoretical maximum value shown in Figure 1 and those of other human powered devices.

The dashed lines in the pulling force plot indicate the rewinding intervals in which there is no input force but the magnet rotor is still rotating due to its inertial mass against electromagnetic damping that is, freewheeling. The generated voltage repeats its cycles with increasing and decreasing amplitude during the pulling and freewheeling phase, respectively. The peak speed of the rotor continuously increased in the following cycles, which can be recognized by the swelling waveforms of the voltage output as additional pulling force was applied on the bobbin. The force applied to the bobbin was also being increased, which is in accordance with the increased speed of the rotor because the electromagnetic damping becomes proportionally larger at higher speed. Table 3 shows the performance in detail.

**Table 3:** The measured performance of the prototype generator.

Pulling Section	Length of Pulled String (mm)	Avg. Pulling Force (N)	Avg. RPM of Magnet Rotor	Period of Time of Pulling (s)	Period of Time of Flywheel (s)
1	129	11.3474	2843	1.3215	1.2201
2	124	12.7651	3650	0.6480	1.2606
3	117	14.9333	3399	0.5789	1.3472
4	128	15.0706	4037	0.5553	1.225
5	127	13.8417	3884	0.5525	1.2822
6	153	18.1973	4640	0.6127	1.2270
7	144	15.6972	4352	0.5660	1.2830
8	124	14.4063	3950	0.5000	1.2610
9	151	17.0400	4879	0.5750	1.1580
10	144	17.1082	1263	0.5880	5.7880

**Figure 9:**
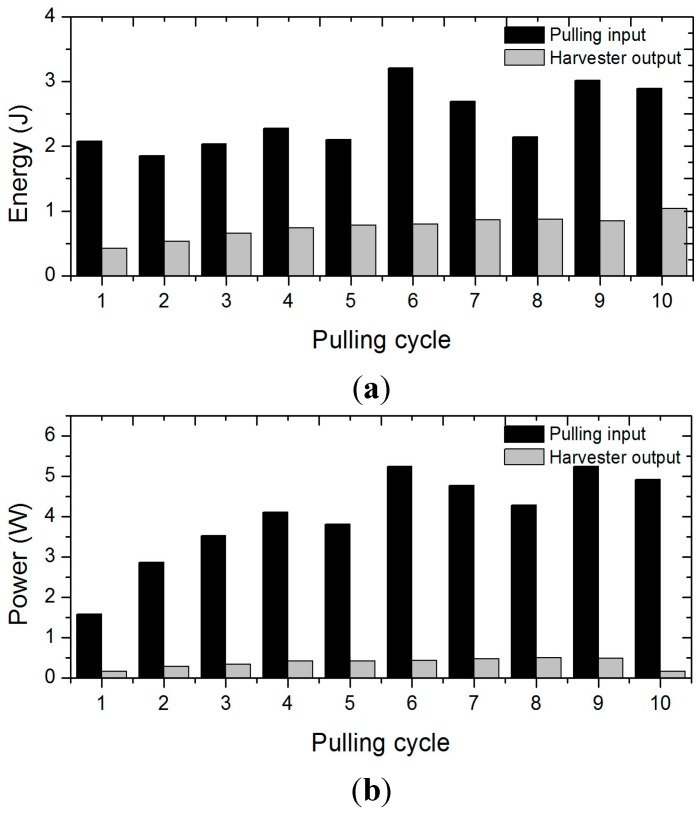
Mechanical pulling input and electrical output of all the cycles. (**a**) Energy; (**b**) Power.

In the 1st pulling cycle, it took a long time for the magnet rotor to start up its rotation and its peak speed was not as high. Consequently, the electricity output showed the lowest power value in the 1st cycle, but significantly increased in the following cycles as shown in Table 4 and Figure 9.

However, it should be noted that the mechanical input also had its lowest value in the 1st cycle and remarkably increased in the following cycles. The mechanical input power, which is far less than its maximum possible value shown in the Figure 1, caused the small electric output during the 1st cycle. Therefore, the efficiency of energy conversion in the 1st cycle is no less than the efficiency in the other cycles.

**Table 4:** Comparison of the mechanical pulling input and electrical voltage output in all cycles.

Pulling Cycle	Input Energy (J)	Output Energy (J)	Avg. Input Power (W)	Avg. Output Power (W)	Max. Input Power (W)	Max. Output Power (W)
1	2.0782	0.4224	1.5735	0.1662	5.0785	0.8100
2	1.8568	0.5301	2.8681	0.2777	7.7463	1.1374
3	2.0360	0.6536	3.5200	0.3393	8.3403	1.3352
4	2.2768	0.7367	4.1039	0.4138	11.6580	1.6248
5	2.1013	0.7766	3.8067	0.4233	10.4961	1.6337
6	3.2099	0.7999	5.2390	0.4348	10.8481	1.6397
7	2.6957	0.8658	4.7635	0.4682	11.8695	1.8293
8	2.1400	0.8698	4.2800	0.4939	11.9620	1.8420
9	3.0142	0.8438	5.2430	0.4869	12.4095	1.8879
10	2.8904	1.0364	4.9174	0.1625	11.1328	1.8333

The sustained rotation, even though it slowed down due to electromagnetic damping, enabled additional pulling to boost its speed while accumulating mechanical energy in the rotor itself. It is important to note that this fulfills the key requirement for the large power production of the pulling limb motion. The enhanced mechanical input power in Table 4 and Figure 9b verified the feasibility of the proposed pulling energy harvester in extracting effective human power by showing that the air-cored coils stator, inertial magnet rotor, and one-way clutched power transmission realized the fast rotation of magnet rotor with consecutive application of the small torque upper limb motion. As a consequence, the maximum electric output went beyond 1.5 W in most cycles and even up to 1.89 W in the 9^th^ pulling cycle. This configuration also contributed to the practical implementation of the device operation employing a finite length of string.

The input, output, and average energy conversion efficiency for the entire operation cycles are shown in Table 5. The input energy, output energy, and output power cover both the pulling and rewinding phases, but the input power is still calculated only for pulling phases for clear indication of human motion power as mentioned before. The developed pulling energy harvester prototype has a notably small size of 50 mm × 32 mm × 43.5 mm and only 0.05 kg weight. Nevertheless, it can generate an average electric power of 0.32 W by converting an average mechanical power of 4.0 W. The average energy conversion efficiency of 30.98% is fairly high considering that those of conventional products employing metal-cored electromagnetic coupling are around 20% [13]. It is also slightly greater than the empirical efficiency of conventional crank generator measured from the controlled experiment under laboratory condition shown in [13], in which the crank rotation is maintained at optimum speed for maximum electromagnetic conversion of the rotational generator. It is worthy to be noted that the crank generator was shown to be better than pulling generator in that experiment [13]. The lower efficiency of the pulling type generator obtained from comparative experiment in the referenced study is caused by the mismatch between the low driving torque and high cogging resistance of the rotor in the pulling experiment. Accordingly, the combination of low-torque pulling motion and low-resistance coreless APMM turned out to be a mechanical impedance matching between human pulling and rotor rotation. Consequently, this proves the high efficiency of the developed prototype human powered generator.

**Table 5:** The input, output, and conversion efficiency.

Mechanical Input		Electrical Output		Conversion Efficiency
Energy (J)	Power (W)	Energy (J)	Power (W)	Ratio (%)
24.30	3.74	7.53	0.32	30.98

## 4. Conclusions/Outlook

This study showed through analytic models for both operations that the upper limb pulling motion had improved human motion economy as well as efficient human-mechanical power transfer than the conventional turning motion. Also, a prototype of the pulling energy harvester was developed by combining an air-cored coil stator, flywheel magnet rotor, and one-way clutched small bobbin to derive effective power from the pulling motion. Its performance and dynamic characteristics verified that the proposed scheme was successful not only in deriving effective human power but also in converting mechanical energy into electric energy by accomplishing higher conversion efficiency than conventional values.

The average output power of 0.32 W with the maximum performance of 1.89 W guarantees the instant powering of most mobile electronic devices. Furthermore, because the theoretical maximum mechanical power (23 W) that could be produced by human pulling motions presented in Figure 1 has not been reached yet, it is likely possible to generate far more electric power by further modification of the developed pulling energy harvester, e.g., by employing a heavier flywheel rotor to engage more human power.

More importantly, the fairly high efficiency (30.98%) of the proposed energy harvester suggests further investigation into the kinetic characteristics of human motions to be harnessed, and more advanced power transmission techniques should bring more improvements in the human-powered system. This system may also be beneficial for wearable devices and human interface devices that accompany active human motions by providing not only self-sustainable power but also an opportunity for unique user experience (UX) [19,20,21].

In addition to the high energy conversion efficiency, there are a few more advantages of the proposed pulling generator as a human powered portable generator. It can make free users’ hands from tight grabbing of chassis and handle of the generator together as is the case with the crank generator. Since the pulling force is applied linearly, users can utilize various limb and body movement, and even different body segments to apply force into it. Furthermore, it can generate electricity by one-hand operation simply by hooking it to a near spot even on the body limb or trunk, which gives benefit to users who are engaged in other daily activity such as phone-call or physical exercise.

In conclusion, it is expected that this study can contribute to realizing not only a truly seamless but also a sustainable mobile environment by providing self-electricity. Future study will be focused on design for comfortable use, low-cost implementation, and scale-up modification of the human-powered generator in favor of sustainable development.
